# Supplementary material for: FASDetect as a machine learning-based screening app for FASD in youth with ADHD
Source: NPJ Digit Med. 2023 Jul 19;6:130. doi: 10.1038/s41746-023-00864-1 (PMC10356778; doi:10.1038/s41746-023-00864-1)
Supplement: Supplementary file 1 — Supplementary Material [file 41746_2023_864_MOESM1_ESM.pdf]

FASDetect as a machine learning-based screening app for FASD in youth with ADHD

Supplementary Figures

Logistic Regression

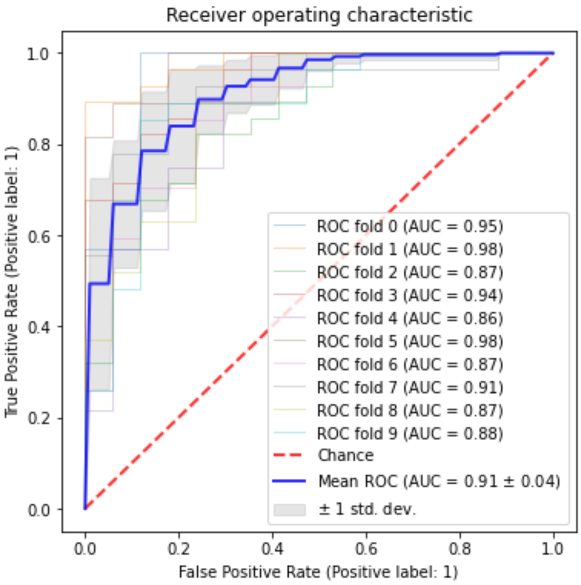

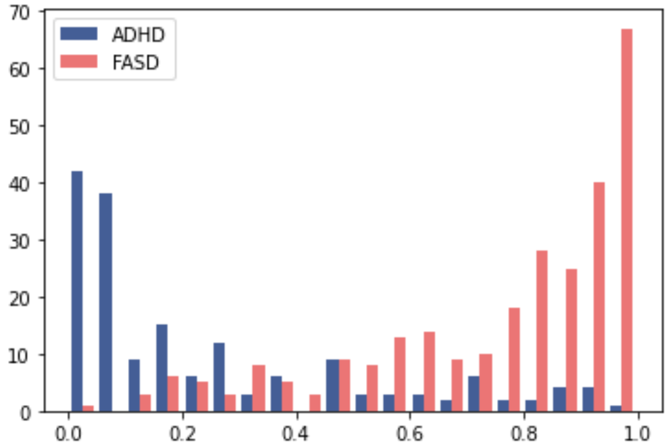

SVM

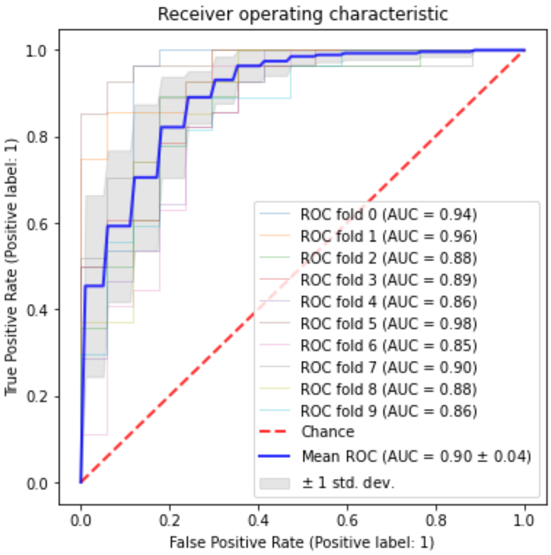

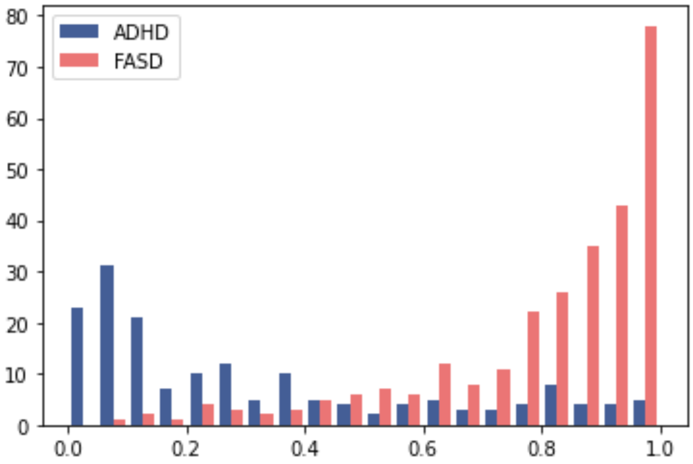

Supplementary Figure 1. ROC curves (left column) and plot of the distribution of the predicted probabilities (right column) for logistic regression (first row) and SVM (second row), based on their application with 13 variables. In the plot of predicted probabilities, the x-axis shows the predicted probability of having FASD and the y-axis the number of actual ADHD/FASD cases for this probability.

RF

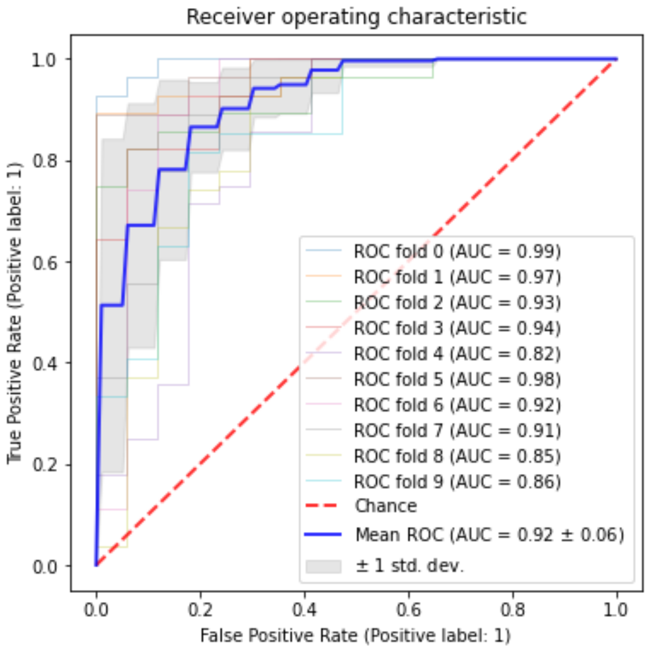

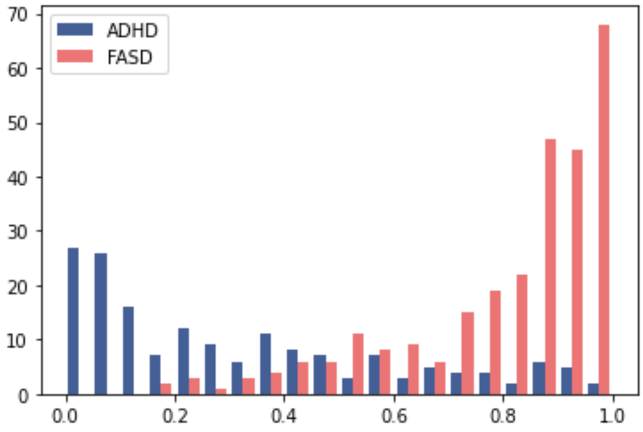

GBDT

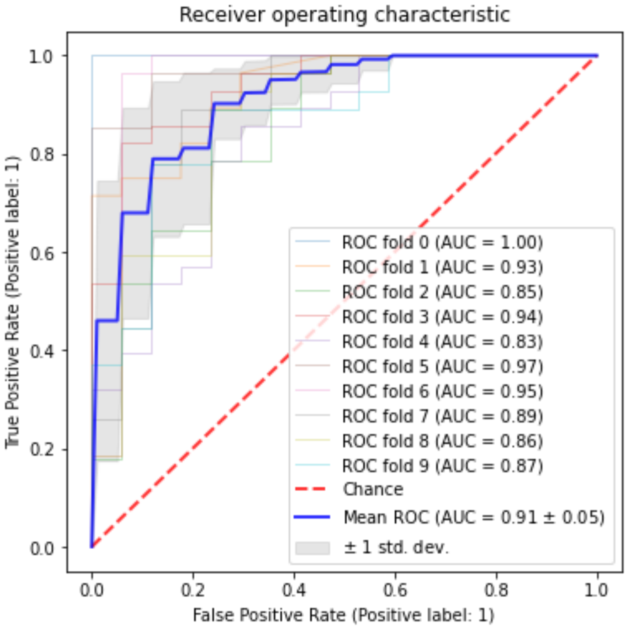

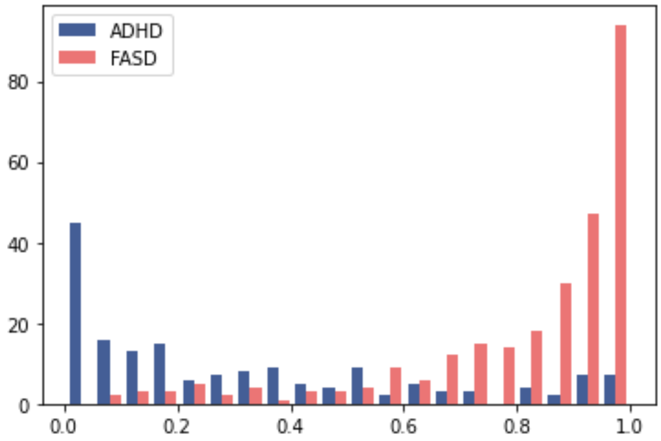

Supplementary Figure 2. ROC curves (left column) and plot of the distribution of the predicted probabilities (right column) for random forests (first row) and GBDT (second row), based on their application with 13 variables. In the plot of predicted probabilities, the x-axis shows the predicted probability of having FASD and the y-axis the number of actual ADHD/FASD cases for this probability.

kNN

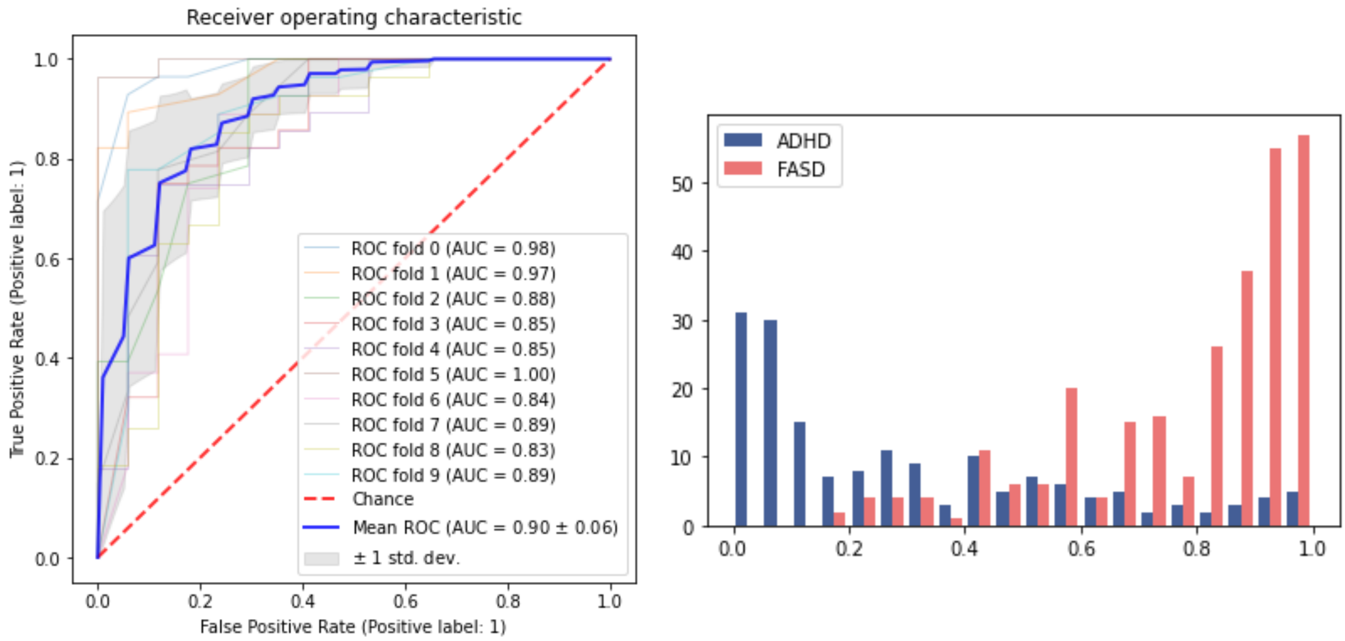

GP

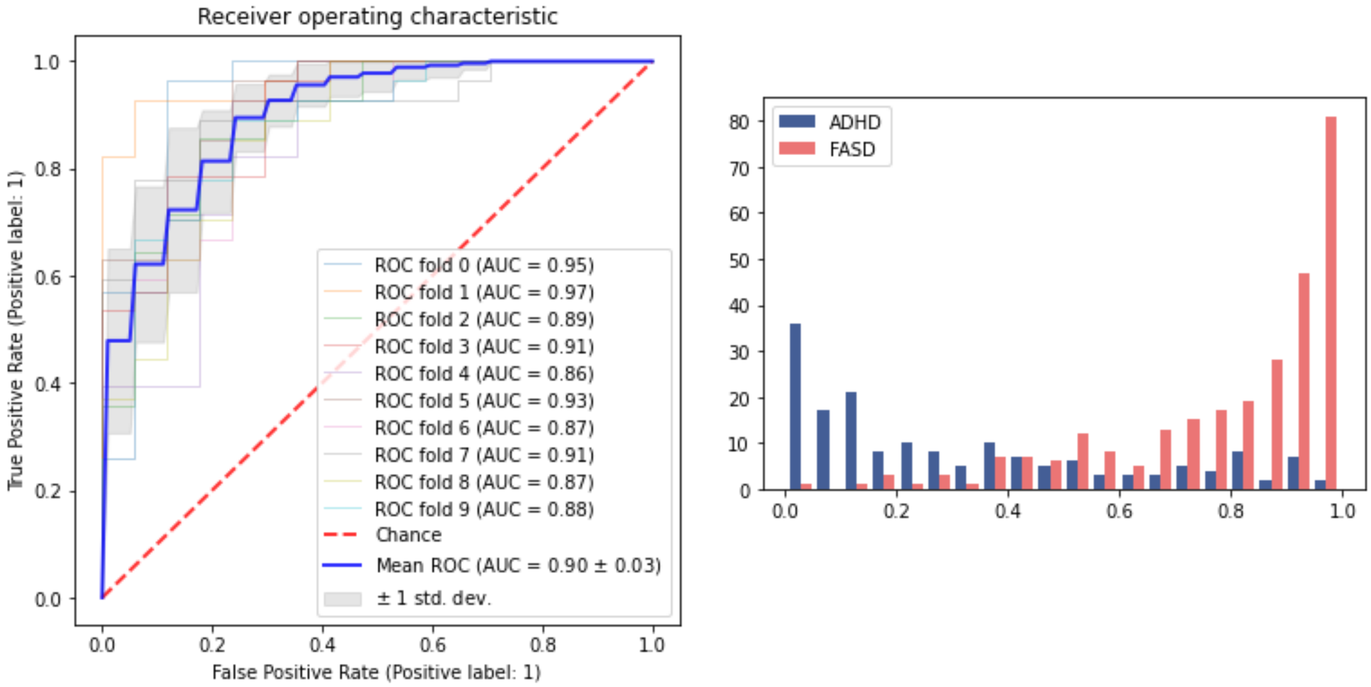

Supplementary Figure 3. ROC curves (left column) and plot of the distribution of the predicted probabilities (right column) for kNN (first row) and GP (second row), based on their application with 13 variables. In the plot of predicted probabilities, the x-axis shows the predicted probability of having FASD and the y-axis the number of actual ADHD/FASD cases for this probability.

LR

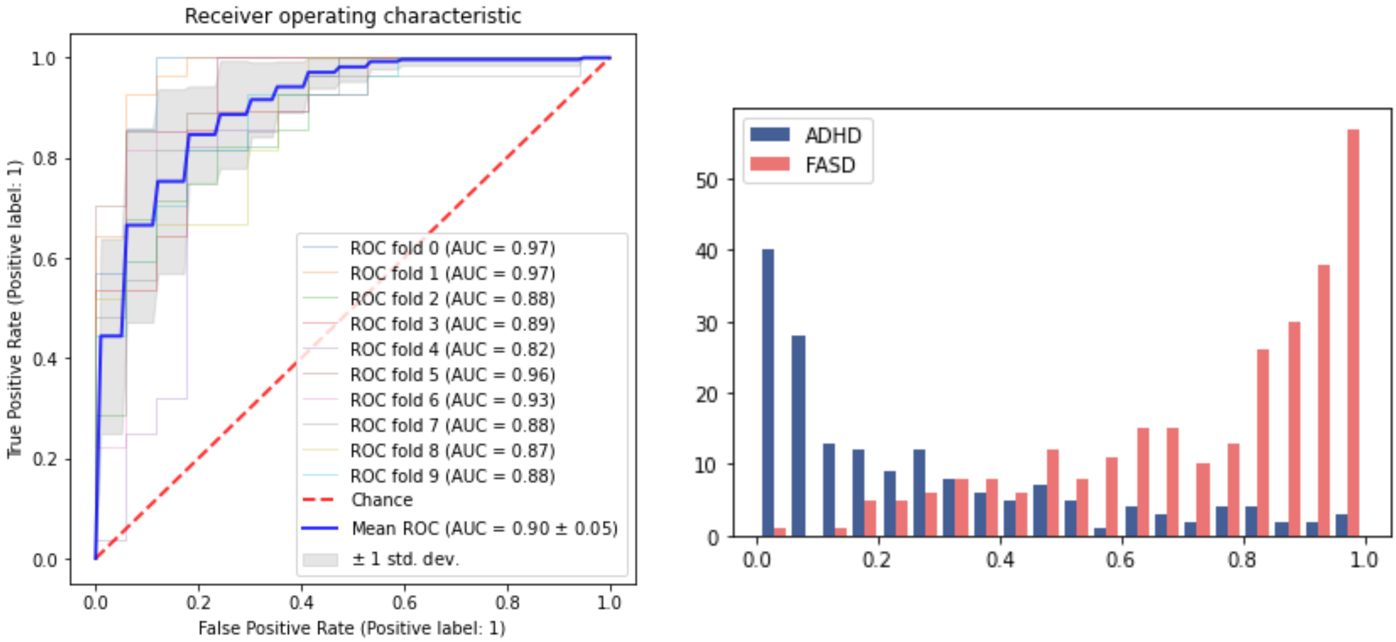

SVM

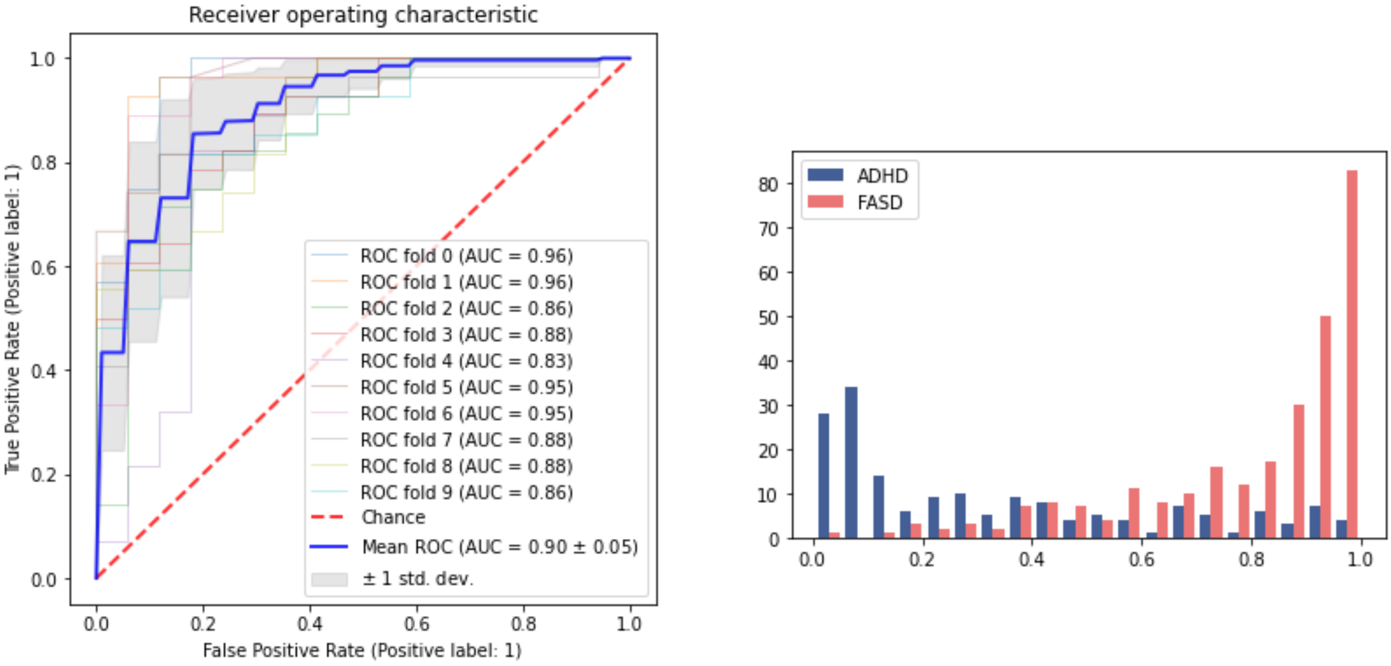

Supplementary Figure 4. ROC curves (left column) and plot of the distribution of the predicted probabilities (right column) for logistic regression (first row) and SVM (second row), based on their application with 6 variables. In the plot of predicted probabilities, the x-axis shows the predicted probability of having FASD and the y-axis the number of actual ADHD/FASD cases for this probability.

RF

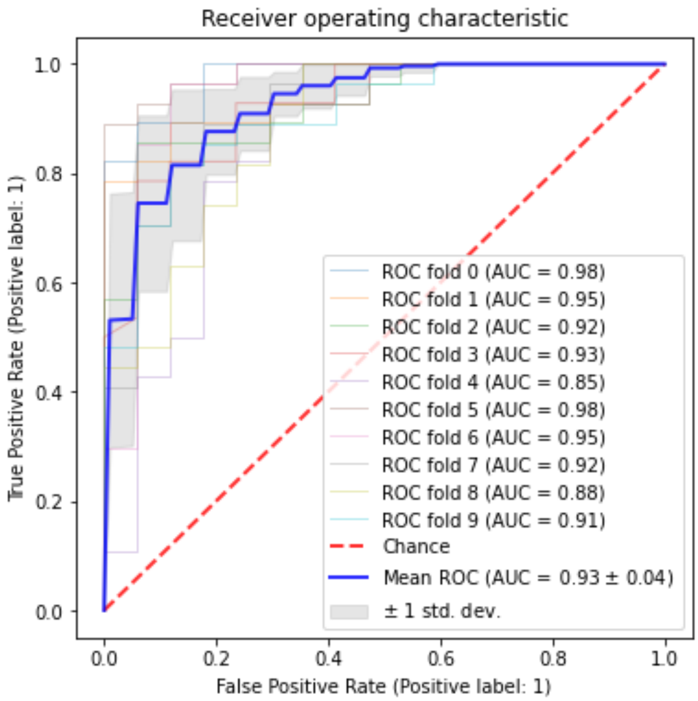

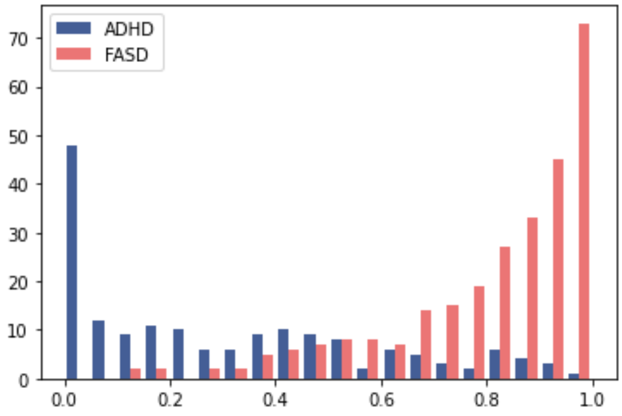

GBDT

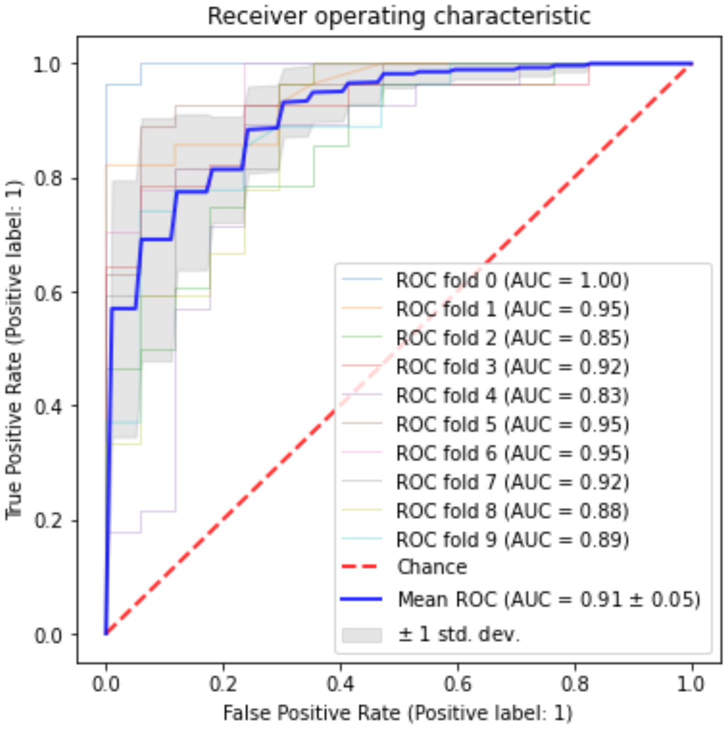

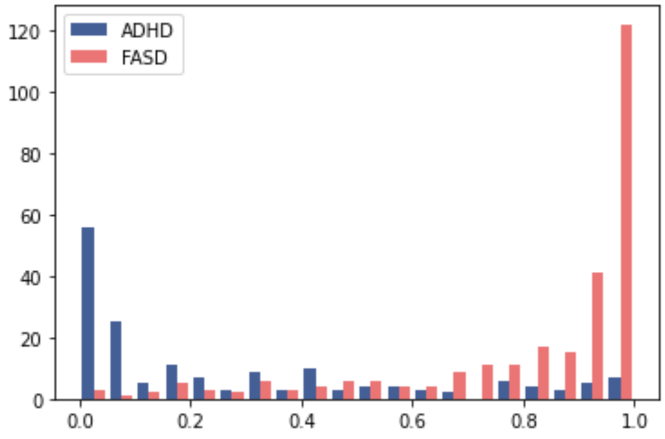

Supplementary Figure 5. ROC curves (left column) and plot of the distribution of the predicted probabilities (right column) for random forests (first row) and GBDT (second row), based on their application with 6 variables. In the plot of predicted probabilities, the x-axis shows the predicted probability of having FASD and the y-axis the number of actual ADHD/FASD cases for this probability.

kNN

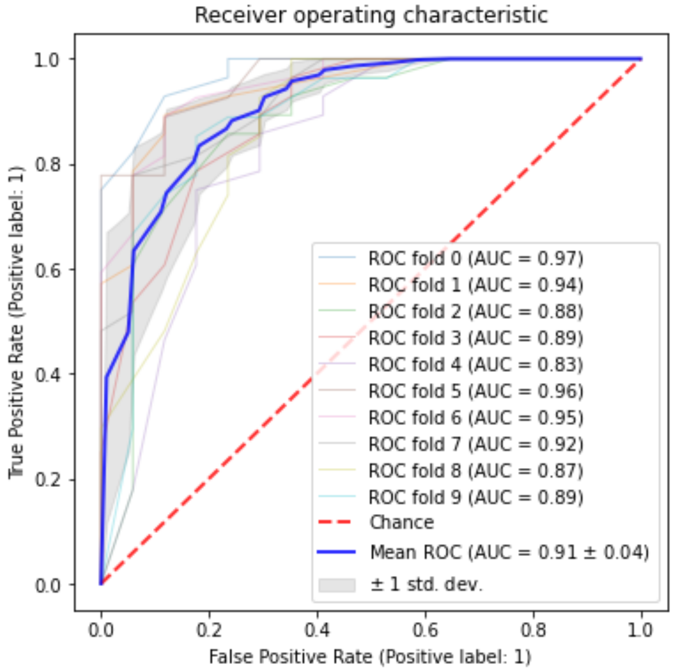

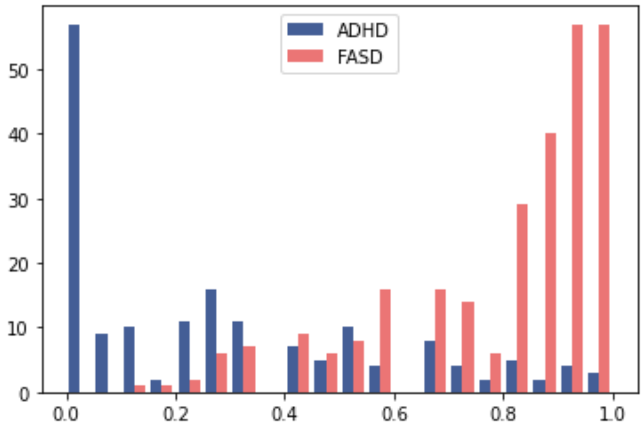

GP

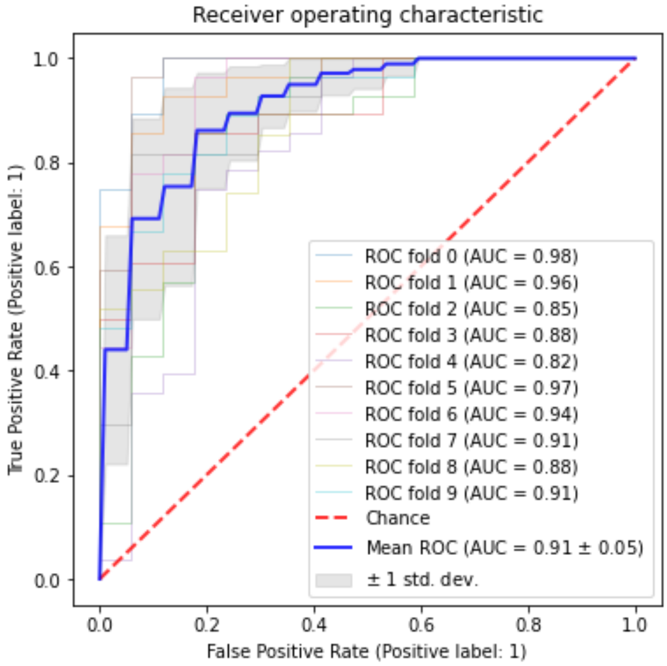

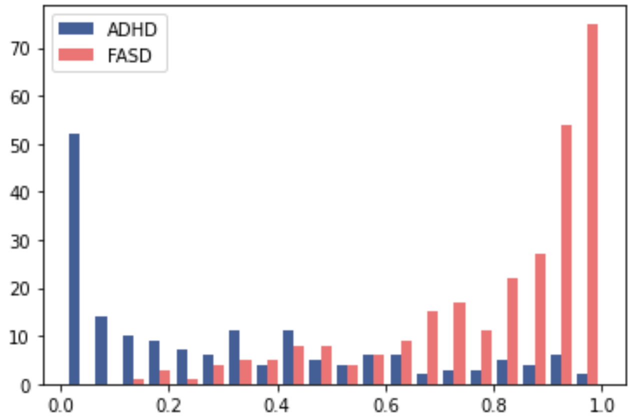

Supplementary Figure 6. ROC curves (left column) and plot of the distribution of the predicted probabilities (right column) for kNN (first row) and GP (second row), based on their application with 6 variables. In the plot of predicted probabilities, the x-axis shows the predicted probability of having FASD and the y-axis the number of actual ADHD/FASD cases for this probability.
