# Supplementary material for: FASDetect as a machine learning-based screening app for FASD in youth with ADHD
Source: NPJ Digit Med. 2023 Jul 19;6:130. doi: 10.1038/s41746-023-00864-1 (PMC10356778; doi:10.1038/s41746-023-00864-1)
Supplement: Supplementary file 2 — Reporting Summary [file 41746_2023_864_MOESM2_ESM.pdf]

nature portfolio

Corresponding author(s): Stefan Konigorski

Last updated by author(s): Jan 28, 2023

Reporting Summary

Nature Portfolio wishes to improve the reproducibility of the work that we publish. This form provides structure for consistency and transparency in reporting. For further information on Nature Portfolio policies, see our Editorial Policies and the Editorial Policy Checklist.

Statistics

For all statistical analyses, confirm that the following items are present in the figure legend, table legend, main text, or Methods section.

n/a	Confirmed	
☐	☒	The exact sample size (*n*) for each experimental group/condition, given as a discrete number and unit of measurement
☐	☒	A statement on whether measurements were taken from distinct samples or whether the same sample was measured repeatedly
☐	☒	The statistical test(s) used AND whether they are one- or two-sided<br>*Only common tests should be described solely by name; describe more complex techniques in the Methods section.*
☐	☒	A description of all covariates tested
☐	☒	A description of any assumptions or corrections, such as tests of normality and adjustment for multiple comparisons
☐	☒	A full description of the statistical parameters including central tendency (e.g. means) or other basic estimates (e.g. regression coefficient) AND variation (e.g. standard deviation) or associated estimates of uncertainty (e.g. confidence intervals)
☒	☐	For null hypothesis testing, the test statistic (e.g. *F*, *t*, *r*) with confidence intervals, effect sizes, degrees of freedom and *P* value noted<br>*Give P values as exact values whenever suitable.*
☒	☐	For Bayesian analysis, information on the choice of priors and Markov chain Monte Carlo settings
☒	☐	For hierarchical and complex designs, identification of the appropriate level for tests and full reporting of outcomes
☐	☒	Estimates of effect sizes (e.g. Cohen's *d*, Pearson's *r*), indicating how they were calculated

Our web collection on statistics for biologists contains articles on many of the points above.

Software and code

Policy information about availability of computer code

Data collection	All Python code used to perform the analyses is publicly available at https://github.com/HIAlab/FASDetect
Data analysis	All Python code used to perform the analyses is publicly available at https://github.com/HIAlab/FASDetect

For manuscripts utilizing custom algorithms or software that are central to the research but not yet described in published literature, software must be made available to editors and reviewers. We strongly encourage code deposition in a community repository (e.g. GitHub). See the Nature Portfolio guidelines for submitting code & software for further information.

Data

Policy information about availability of data

All manuscripts must include a data availability statement. This statement should provide the following information, where applicable:

- Accession codes, unique identifiers, or web links for publicly available datasets
- A description of any restrictions on data availability
- For clinical datasets or third party data, please ensure that the statement adheres to our policy

The data analyzed in this study represents sensitive identifying clinical data of patients with ADHD and patients visiting the FASD specialist center at the Campus Charité Virchow of the Charité – Universitätsmedizin, and cannot be made available.

Human research participants

Policy information about studies involving human research participants and Sex and Gender in Research.

Reporting on sex and gender	Sex was considered in the study and in the main analysis.
Population characteristics	Tables 1-2 give an overview of the main characteristics of the n = 445 FASD and ADHD patients, which included 159 female (mean age at initial presentation, 9.6 years [range, 0.2–18.8 years]) and 286 male (mean age at initial presentation, 8.9 years [range, 0.1–19.0 years]) patients. 139 of the FASD patients had a FAS diagnosis, 127 had a pFAS diagnosis and 9 patients were diagnosed with ARND. 170 patients belonged to the ADHD group (31 female; mean age at initial presentation, 8.7 years [range, 3.7–16.8 years]; 139 male; mean age at initial presentation, 8.4 years [range, 2.3–15.7 years]) and 275 patients belonged to the FASD group (128 female; mean age at initial presentation, 9.9 years [range, 0.2–18.8 years]; 147 male; mean age at initial presentation, 9.4 years [range, 0.1–19.0 years]). There were very low pairwise correlations between these variables, with the exception of head circumference and birth length (Pearson correlation coefficient of 0.57).
Recruitment	This study was conducted at the outpatient unit of the department of child and adolescent psychiatry at the Campus Charité Virchow of the Charité Universitätsmedizin Berlin, Germany. The sample for the analysis was selected to allow a comparison of patients with a diagnosis of ADHD with patients with a diagnosis of FASD. More specifically, a group of consecutively assessed patients with a clinical diagnosis of ADHD without FASD and a group of patients with an expert diagnosis of FASD (with or without comorbid ADHD) was compared. Altogether, 694 patients with ADHD symptoms were identified consecutively from the general patient pool being potentially eligible for the study. 256 of the 694 ADHD patients had a confirmed FASD diagnosis and therefore were excluded from the ADHD pool. Further, 141 patients were excluded from the ADHD group due to an unconfirmed ADHD diagnosis; 58 because they had a suspected but not confirmed FASD diagnosis; 37 due to other severe medical, psychiatric, or neurological conditions; and 32 patients were excluded because patient records were unavailable. This yielded in total 170 patients in the ADHD group. The consecutively enrolled FASD group was recruited from the specialist center and consisted of 275 youth, including 129 FASD patients with comorbid ADHD and 146 patients without comorbid ADHD diagnosis. These 275 patients included most of the 256 FASD patients from the general patient pool. See also Figure 1 for an illustration of the two study groups.
Ethics oversight	Charité – Universitätsmedizin Berlin

Note that full information on the approval of the study protocol must also be provided in the manuscript.

Field-specific reporting

Please select the one below that is the best fit for your research. If you are not sure, read the appropriate sections before making your selection.

☒ Life sciences ☐ Behavioural & social sciences ☐ Ecological, evolutionary & environmental sciences

For a reference copy of the document with all sections, see nature.com/documents/nr-reporting-summary-flat.pdf

Life sciences study design

All studies must disclose on these points even when the disclosure is negative.

Sample size	We performed a retrospective chart review study. All available data was included in the study, and no sample size calculation was performed. We anticipated that the data was sufficienctly large to achieve a good accuracy in the prediction algorithms (also since it is bigger than comparable studies in the literature) and the results support our assumption.
Data exclusions	This study was conducted at the outpatient unit of the department of child and adolescent psychiatry at the Campus Charité Virchow of the Charité Universitätsmedizin Berlin, Germany. The sample for the analysis was selected to allow a comparison of patients with a diagnosis of ADHD with patients with a diagnosis of FASD. More specifically, a group of consecutively assessed patients with a clinical diagnosis of ADHD without FASD and a group of patients with an expert diagnosis of FASD (with or without comorbid ADHD) was compared. Altogether, 694 patients with ADHD symptoms were identified consecutively from the general patient pool being potentially eligible for the study. 256 of the 694 ADHD patients had a confirmed FASD diagnosis and therefore were excluded from the ADHD pool. Further, 141 patients were excluded from the ADHD group due to an unconfirmed ADHD diagnosis; 58 because they had a suspected but not confirmed FASD diagnosis; 37 due to other severe medical, psychiatric, or neurological conditions; and 32 patients were excluded because patient records were unavailable. This yielded in total 170 patients in the ADHD group. The consecutively enrolled FASD group was recruited from the specialist center and consisted of 275 youth, including 129 FASD patients with comorbid ADHD and 146 patients without comorbid ADHD diagnosis. These 275 patients included most of the 256 FASD patients from the general patient pool. See also Figure 1 for an illustration of the two study groups.
Replication	We performed internal validation through the training-validation-test data setup.
Randomization	We focused on the development of prediction models, not on hypothesis testing, but still built the prediction models based on assumed causal graphs
Blinding	Blinding was not done since in the data collection, we performed careful review of all chart entries and diagnosis to avoid data errors and misdiagnoses.

Reporting for specific materials, systems and methods

We require information from authors about some types of materials, experimental systems and methods used in many studies. Here, indicate whether each material, system or method listed is relevant to your study. If you are not sure if a list item applies to your research, read the appropriate section before selecting a response.

Materials & experimental systems

n/a	Involved in the study
☒	☐ Antibodies
☒	☐ Eukaryotic cell lines
☒	☐ Palaeontology and archaeology
☒	☐ Animals and other organisms
☒	☐ Clinical data
☒	☐ Dual use research of concern

Methods

n/a	Involved in the study
☒	☐ ChIP-seq
☒	☐ Flow cytometry
☒	☐ MRI-based neuroimaging
